# Energy-based jamming pattern open set recognition via spiking wavelet transformer

**DOI:** 10.1371/journal.pone.0325381

**Published:** 2025-06-26

**Authors:** Xincheng Zhong, Junbiao Cui, Zhuangwei Ji

**Affiliations:** 1 Department of Computer Science, Changzhi University, Changzhi, Shanxi, China; 2 Key Laboratory of Computational Intelligence and Chinese Information Processing of Ministry of Education, School of Computer and Information Technology, Shanxi University, Taiyuan, Shanxi, China; University of Essex Faculty of Science and Engineering, UNITED KINGDOM OF GREAT BRITAIN AND NORTHERN IRELAND

## Abstract

Jamming pattern recognition (JPR) is critical in modern communication systems, including civil, and industrial applications, where complex and unpredictable jamming patterns (JPs) frequently disrupt normal operations. Accurately identifying known JPs while detecting unknown JPs is essential for maintaining robust communication. This paper tackles the jamming pattern open set recognition (JP-OSR) challenge by proposing a novel framework that integrates three key components: Spiking Wavelet Transformer (SWT), Hyperspherical Maximum Class Separation (MCS), and an energy-based model with an adaptive threshold. The framework begins by extracting high-resolution time-frequency features through the SWT, effectively capturing the intricate temporal and spectral patterns of jamming signals. These features are then aligned in a hyperspherical space using MCS to maximize class separation and improve inter-class distinctions. To address the variability in feature distributions across different Jamming-to-Signal Ratios (JSR) and JPs, the proposed energy-based model dynamically adjusts the threshold, enabling accurate recognition of both known and unknown jamming types. Simulation experiments have validated the effectiveness and superiority of the proposed method for JP-OSR.

## Introduction

In the realm of contemporary electronic systems, the ongoing interaction between different systems involves the continuous development of interference and mitigation techniques. These approaches are growing increasingly advanced, leading to the emergence of innovative and more assertive JPs. The recognition of such patterns plays a crucial role in mitigating interference, as it directly impacts the robustness and security of communication systems [1,2]. This is particularly critical in complex and dynamic electromagnetic environments where traditional recognition methods often struggle to adapt.

To ensure communication system robustness, jamming recognition techniques must not only precisely identify known patterns but also detect and adapt to previously unseen, unknown jamming types. This dual requirement highlights the necessity for OSR approaches in jamming pattern identification [3,4]. Such approaches expand the recognition space to include an unknown category $X_{\text{unknown}}$, enabling models to handle new and potentially harmful patterns without prior training data.

Traditional jamming recognition methods are largely divided into two categories: manually crafted feature extraction techniques and deep learning-based approaches. The former, often based on statistical or signal processing methods such as Fourier or wavelet transforms, struggles with complex multi-dimensional signals and overlapping JPs [5,6]. The latter, including convolutional neural networks (CNNs) and recurrent architectures, has made significant strides in handling structured data. However, these models typically operate in closed-set scenarios, limiting their applicability in open-set environments where unknown jamming types frequently emerge [7,8].

Moreover, effective feature extraction remains a core challenge. Current feature extraction techniques are constrained by the dimensionality of the input data, focusing on specific features such as spectral aggregation, frequency domain statistics, or signal entropy [5,6]. While these features are suitable for certain tasks, they fail to generalize across different jamming types, particularly when data is sparse or non-stationary. Additionally, the reliance on a single feature extraction strategy, such as average frequency suppression or energy compaction, cannot adequately capture the intricate dynamics of modern jamming signals.

With the rise of event-driven vision tasks, Spiking Neural Networks (SNNs) have gained popularity for their energy efficiency, and integrating Transformer architectures with SNNs has shown promise for accuracy improvement; however, conventional self-attention struggles to capture high-frequency patterns such as moving edges and pixel-level brightness changes. To address the challenge of learning high-frequency features, we propose the Spiking Wavelet Transformer (SWT), an attention-free architecture that leverages the sparse wavelet transform for efficient spatial-frequency learning in a spike-driven manner.

Recent advancements in OSR have explored probabilistic models, energy-based methods, and generative techniques to address the challenges posed by unknown samples. Energy-based methods, for instance, define a scalar energy score to measure a sample’s compatibility with known categories, enabling efficient detection of out-of-distribution (OOD) samples [4,9]. Other approaches, such as generative adversarial networks (GANs) and variational autoencoders (VAEs), aim to synthesize plausible representations of unknown patterns to enhance recognition robustness [10,11]. However, these methods often require significant computational resources and fail to scale effectively to high-dimensional, multi-modal signals [12]. Furthermore, as highlighted in [13], a well-performing closed-set classifier can also excel in OSR tasks. Accordingly, we employ the MCS approach to maximize the angular margins between classes, facilitating compact clustering of known classes and effective rejection of unknown classes.

Specifically in JPR, methods based on signal reconstruction convolutional neural networks (SRCNNs) have gained attention for their ability to extract multi-scale features. These models demonstrate promise in identifying known jamming types, but their performance degrades when faced with new, unseen patterns due to their reliance on fixed feature representations [14]. Open-set approaches, such as those utilizing hyperspherical prototypes or hybrid architectures, have shown potential in bridging this gap by dynamically adapting to both known and unknown categories [9,15].

In this study, we propose a novel framework for JPR, which integrates the SWT [14] for feature extraction, MCS [8] for classification, and an energy-based model [4] for JP-OSR. The SWT integrates spiking neural dynamics with wavelet-based spectral analysis, offering a biologically inspired, energy-efficient mechanism to capture both temporal and frequency-domain characteristics of jamming signals [5,11]. Meanwhile, the MCS module aligns features onto a hypersphere, ensuring uniform class separation and enhancing the ability to differentiate between known and unknown patterns [7,9].

Our contributions are as follows:

1. Enhanced Feature Representation: The proposed framework integrates a multi-resolution feature extraction approach, leveraging the SWT to effectively capture both time-frequency characteristics and dynamic variations of non-stationary and overlapping jamming signals.
2. Improved Classification Accuracy: By incorporating the MCS mechanism, the framework ensures precise alignment of class prototypes on a hypersphere, enabling robust classification boundaries for known JPs.
3. Efficient Open-Set Detection: The integration of an energy-based detection module provides a reliable method for identifying unknown jamming signals, reducing dependence on handcrafted features and enhancing adaptability to complex, real-world electromagnetic environments.

The remainder of this paper is organized as follows. We begin by reviewing related work. We then introduce our open-set recognition model designed to identify unknown jamming patterns, followed by a description of the proposed methods. The subsequent section presents the simulation experiments and results. Finally, we conclude the paper with a discussion of limitations and future research directions.

## Related work

### Open-set recognition (OSR)

OSR focuses on distinguishing between known and unknown categories, particularly in scenarios where unknown samples coexist with predefined classes. Traditional OSR approaches can be categorized more accurately as follows:

1. Distance-Based Models [16–18]:

These models leverage feature space distances, such as Euclidean distance or cosine similarity, to separate known and unknown samples. Examples include nearest neighbor approaches and models that define thresholds based on class centroids or prototypes.

2. Reconstruction-Based Models [19,20]:

These methods utilize reconstruction errors from generative models, such as autoencoders or variational autoencoders, to identify unknown samples. Known classes are expected to have lower reconstruction errors compared to unknown ones.

3. Probability-Based Models [21,22]:

These approaches estimate class probabilities using techniques like Softmax. However, Softmax-based models often struggle to differentiate between in-distribution (ID) and out-of-distribution (OOD) samples due to their overconfidence in predictions.

In this work, we adapt energy-based models [4,23], traditionally designed for OOD detection, to JP-OSR tasks. By leveraging their capability to establish a clear boundary between known and unknown classes, we introduce a novel approach for jamming pattern recognition, improving the robustness and scalability of OSR in real-world scenarios.

### JPs recognition and feature extraction

Recognizing and extracting features from jamming signals are critical tasks in complex electromagnetic environments, especially under adversarial conditions. Existing methods can be broadly categorized as follows:

1. Traditional Spectral Analysis [24]: Techniques like Short-Time Fourier Transform (STFT) and Discrete Wavelet Transform (DWT) analyze signals in the time-frequency domain and effectively capture local signal characteristics. However, they struggle to handle overlapping or dynamic JPs, particularly when dealing with previously unseen jamming types.
2. Deep Learning-Based Approaches [25,26]: Neural networks, such as Convolutional Neural Networks (CNNs) and Long Short-Term Memory networks (LSTMs), automate feature learning and excel in closed-set classification tasks. However, these methods often fail to generalize to open-set scenarios, where novel jamming types emerge, and they lack effective temporal modeling for highly dynamic signals.

The proposed SWT addresses the limitations of both traditional spectral analysis and deep learning-based approaches by integrating wavelet-based frequency decomposition with the temporal dynamics of spiking neurons. By combining time-frequency analysis and biologically inspired dynamics, SWT enables robust feature extraction for complex JPs. Specifically, it captures both fine-grained local frequency patterns and global temporal structures, while introducing sparse, event-driven activations through spiking neurons to enhance energy efficiency and improve robustness, particularly for non-stationary or high-dimensional signals.

Moreover, SWT seamlessly integrates with open-set recognition modules, allowing for efficient detection and classification of both known and unknown jamming signals. This unified framework ensures adaptability to dynamic electromagnetic environments, making it a highly effective solution for real-world applications.

## Open-set recognition model for unknown JPs

The recognition of JPs is a critical task in complex electromagnetic environments, particularly in highly dynamic and challenging scenarios. This problem entails identifying jamming types employed by adversaries, even when these types are not previously encountered. In an open-set recognition scenario, the recognition space not only includes known jamming categories but also allows for the potential existence of new and unknown types of adversarial jamming. These novel patterns are considered to belong to an unknown set, $X_{\text{unknown}}$.

The challenge with recognizing such unknown JPs lies in the lack of prior knowledge or pre-existing information about these patterns. Within this open-set framework, the set of known jamming categories $J_{\text{known}}=\{J_{1},J_{2},\ldots ,J_{C}\}$ is predefined, but new JPs may emerge that do not fit into any of the existing classes. To accommodate this, the recognition system must dynamically account for an unknown set of JPs, denoted as $J_{\text{unknown}}=\{J_{C+1}\}$.

In real-world applications, continuous signal acquisition introduces additional challenges. The jamming signals from adversarial sources may interact and overlap with other signals in the environment, creating uncertainty in recognition. For adversarial JPs that vary rapidly and only persist for a short duration, immediate identification is often impractical. However, these transient patterns are generally not critical for long-term situational awareness and can often be disregarded in immediate analysis.

On the other hand, persistent or recurring JPs that remain unrecognized should be prioritized for classification. These patterns, if identified as belonging to the unknown set, can be recorded and stored for further analysis and adaptation of the recognition system. By treating these patterns as part of $J_{\text{unknown}}$, the system enhances its ability to adapt to evolving threats.

To address the limitations of traditional jamming pattern recognition systems, this work builds upon existing research to propose an extended OSR framework for jamming classification. The two-step process ensures that:

1. A binary decision first determines whether a signal belongs to the known set $X_{\text{known}}$ or the unknown set X_unknown_.
2. For jamming signals identified as part of $X_{\text{known}}$, classification is performed within the predefined categories. For jamming signals flagged as $X_{\text{unknown}}$, the focus shifts to adaptive learning and storage of the new patterns for subsequent identification.

## Methods

**Fig 1:**
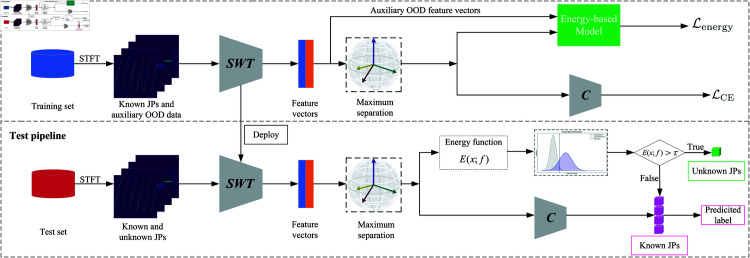
The overall structural flowchart of the proposed method.

The proposed framework for JP-OSR consists of a training phase and a testing phase, seamlessly integrating feature extraction, classification, and unknown class detection, as illustrated in Fig 1.

During training, the known JPs and auxiliary OOD data are transformed using STFT and then processed by the SWT to extract informative feature representations. These features are projected onto a hypersphere for maximum class separation, ensuring uniform and distinct class distributions. The framework employs a dual-loss optimization strategy: cross-entropy loss trains the classifier for precise recognition of known JPs, while an energy-based loss leverages auxiliary OOD data to establish a robust energy boundary for unknown detection.

In the testing phase, the same preprocessing and feature extraction steps are applied to both known and unknown JPs. The energy function evaluates each test sample, and those exceeding the predefined energy threshold are classified as unknown. For samples below the threshold, the classifier assigns them to one of the predefined known categories. This unified approach enables efficient detection and classification of both known and unknown JPs, addressing the challenges of dynamic and complex electromagnetic environments with improved robustness and adaptability.

### Proposed data preprocessing techniques

The raw time-domain waveforms of the sampled jamming signals contain excessive data and redundancy, making them unsuitable for direct training of neural networks. Meanwhile, spectrograms lack temporal information, limiting their effectiveness for jamming pattern recognition when used alone. Therefore, this study leverages time-frequency images generated by Short-Time Fourier Transform (STFT) as input features for JP-OSR, combining both spectral and temporal characteristics to enhance recognition performance. By segmenting the raw signals into fixed-length windows and applying a Hanning window function to each segment, the STFT generates a time-frequency matrix:

$$X(t,f)=\sum_{n=0}^{N-1}x[n]\cdot w[n-t]\cdot e^{-j2\pi fn}.$$ (1)

where *X*(*t*,*f*) represents the time-frequency distribution, *w*[*n*] is the window function, and *N* is the segment length. This matrix captures both temporal and spectral characteristics of the signal, making it suitable for subsequent processing.

To further enhance interpretability, the time-frequency matrix is normalized to amplify feature contrast:

$$X_{\text{norm}}(t,f)=\frac{\left|X(t,f)|-\min(|X(t,f)|\right)}{\max(|X(t,f)|)-\min(|X(t,f)|)}.$$ (2)

The normalized matrix is then mapped to a pseudo-color heatmap using a defined color scale, transforming it into a visual representation that emphasizes differences across frequency bands. This processed time-frequency image provides a robust foundation for feature extraction and signal classification.

### Spiking wavelet transformer (SWT) for feature extraction

**Fig 2:**
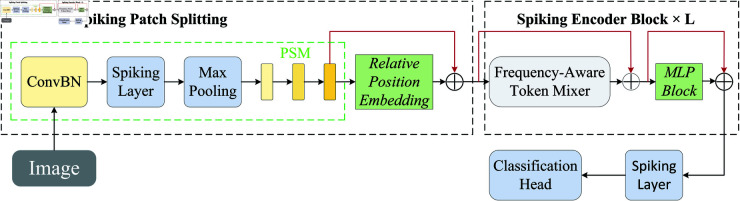
The overview of SWT.

In this framework, the SWT serves as the primary feature extractor, and its specific structural diagram is shown in Fig 2.

The SWT architecture is composed of three main components: the Spiking Patch Splitting (SPS) module, Spiking Encoder Blocks, and a linear classification head. The SPS module integrates convolutional layers (ConvBN), spiking layers, and max pooling operations to enhance frequency perception while preserving spatiotemporal relationships via relative position embedding. The Spiking Encoder Blocks further refine global features through frequency-aware token mixing (FATM) and MLP operations, ensuring compatibility with neuromorphic hardware. FATM serves as the cornerstone of the SWT, integrating three parallel branches: a wavelet-based frequency learner for capturing time-frequency domain features, a convolutional spatial learner for extracting spatial characteristics, and a pointwise convolutional channel mixer for aggregating cross-channel information. This architecture enables the effective decoupling and fusion of spatial and frequency information, enhancing the model’s ability to perceive high-frequency visual components. To further optimize efficiency, SWT employs trinary spike encoding and block-diagonal modular weight matrices, significantly reducing parameter count and computational complexity. Additionally, the incorporation of a membrane potential residual connection strategy bolsters cross-layer signal transmission, maintaining both the biological plausibility and computational efficiency inherent to spike-driven computation. Finally, the linear classification head efficiently maps the extracted features to classification outputs. Due to the requirements of this study, we will not utilize its classification head and will only employ the first two modules for feature extraction.

SWT demonstrates several advantages that make it a powerful tool for signal processing tasks:

1. Improved Frequency Representation: The frequency-aware token mixing mechanism (FATM) enhances the model’s ability to extract and utilize frequency characteristics, critical for processing time-frequency signals.
2. Neuromorphic Efficiency: The exclusive reliance on convolutional and MLP operations reduces computational complexity, making the architecture highly compatible with neuromorphic hardware for low-power applications.
3. Comprehensive Feature Learning: The Frequency Learner module effectively captures multi-scale spectral features using block-diagonal matrix operations, enabling a thorough analysis of signal characteristics.
4. Hybrid Design: By combining spiking neural networks (SNNs) with transformers, SWT capitalizes on the low-energy consumption of SNNs while benefiting from the superior representational power of transformers.

SWT is particularly well-suited for JPR tasks due to its exceptional ability to capture spectral features through its frequency-aware design. The integration of temporal modeling ensures that dynamic variations in jamming signals are retained and exploited for classification. Furthermore, the hardware-friendly design makes SWT ideal for real-time deployment in resource-constrained environments, such as jamming detection and classification systems. Its combination of efficiency, accuracy, and adaptability positions it as a promising solution for JPR and analysis.

### Maximum class separation (MCS) as inductive bias in one matrix

This paper introduces MCS as a foundational geometric inductive bias for classification tasks. Unlike conventional methods that jointly learn class embeddings and feature representations, MCS employs a pre-constructed matrix *P* to encode maximally separated class vectors on a hypersphere. By fixing *P*, the model simplifies the optimization process, decouples class representation learning from feature alignment, and ensures consistent class separation throughout training. This approach not only enhances decision boundaries but also imposes natural geometric constraints through the hyperspherical arrangement of *P*, resulting in a more structured and discriminative feature space. More importantly, separation does not require optimization but can be resolved in closed form prior to training and integrated into the network, thereby effectively enhancing the model’s efficiency.

#### Fixed class representation matrix.

The fixed class representation matrix *P* is the backbone of the MCS framework. It is designed with the following key properties:

1. Dimensionality: $P\in {\mathbb{R}}^{(C-1)\times C}$, where *C* represents the number of classes, and *C*–1 represents the embedding dimensionality.

2. Unit Norm Constraint: Each column of *P*, corresponding to a class vector *P*_*c*_, is normalized to unit length:

$$\|P_{c}\|=1,\;\forall c\in \{1,2,\ldots ,C\}.$$ (3)

3. Equal Pairwise Similarity: All class vectors maintain uniform cosine similarity:

$$P_{i}\cdot P_{j}=\text{constant},\;\forall i\neq j.$$ (4)

4. Zero-Centered Distribution: The mean of all class vectors is zero, ensuring symmetrical distribution:

$$\sum_{c=1}^{C}P_{c}=0.$$ (5)

**Fig 3:**
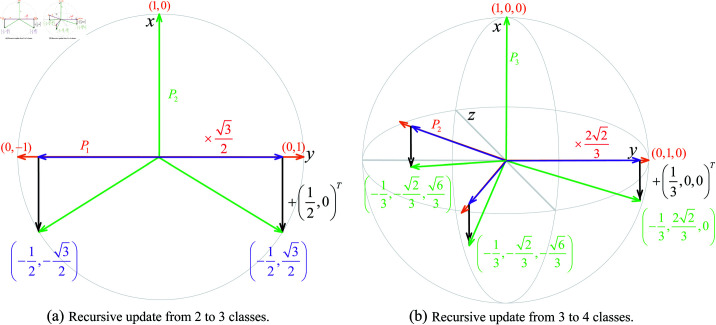
Separation in closed form through recursion. (a) illustrates the process of obtaining maximally separated vectors for three classes on ${\text{S}}^{1}$, starting from two class vectors on a line. Similarly, (b) demonstrates the step from three to four classes on ${\text{S}}^{2}$.

To achieve these properties, *P* is recursively constructed, embedding each new class vector orthogonal to the current subspace. This recursive method guarantees that the new vectors maintain maximum separation on a hypersphere. The closed-form separation recursive process is shown in Fig 3.

#### Feature projection.

After extracting features using SWT, each input ${𝐱}_{i}$ is transformed into a (*C*–1)-dimensional feature embedding ${\hat{𝐱}}_{i}$ using a feature mapping function Φ(·):

$${\hat{𝐱}}_{i}=\Phi ({𝐱}_{i}),\;{\hat{𝐱}}_{i}\in {\mathbb{R}}^{C-1}.$$ (6)

The classification logits *o*_*i*_ are then computed via the dot product of ${\hat{𝐱}}_{i}$ with the pre-constructed matrix *P*:

$$o_{i}=P^{⊤}{\hat{𝐱}}_{i},\;o_{i}\in {\mathbb{R}}^{C}.$$ (7)

This process aligns each feature vector ${\hat{𝐱}}_{i}$ with the predefined directions in *P*, with each logit *o*_*i*,*c*_ representing the similarity of the feature vector to a specific class.

#### Optimization objective.

The network is trained using a cross-entropy loss ${ℒ}_{\text{CE}}$, which ensures that the feature vectors are aligned with the correct class vectors:

$${ℒ}_{\text{CE}}=-\frac{1}{N}\sum_{i=1}^{N}\log\frac{e^{o_{i,y_{i}}}}{\sum_{j=1}^{C}e^{o_{i,j}}}.$$ (8)

where *y*_*i*_ denotes the ground truth label for ${𝐱}_{i}$, and *N* is the number of training samples.

This loss minimizes the discrepancy between the network output and the correct class, implicitly driving ${\hat{𝐱}}_{i}$ to align with the target class vector $P_{y_{i}}$.

### Energy-based JP-OSR

The MCS framework can be extended for open set detection tasks by incorporating an energy-based model. The energy score for a sample is computed from the classification logits: The energy score *E* acts as a compact measure of uncertainty or confidence in the input belonging to the known classes:

$$E({𝐱}_{i})=-T\cdot \log\sum_{j=1}^{C}e^{(P^{⊤}{\hat{𝐱}}_{i}{)}_{j}/T}.$$ (9)

Known samples tend to have lower energy scores because their logits *o*_*i*_ align with the trained class distributions. Unknown samples often produce dispersed or inconsistent logits, leading to higher energy scores, making E>θ a reliable indicator of unknown inputs. Algorithm 1 illustrates how we apply the energy-based model for JP-OSR.

**Algorithm 1.** Self-adaptive energy-based JP-OSR algorithm.

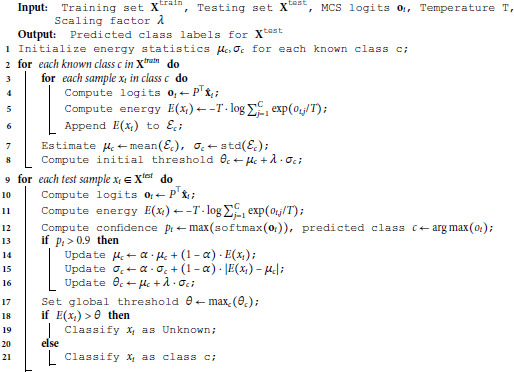



For in-distribution(ID) samples, the energy score *E*(*x*) should be minimized as much as possible. For out-of-distribution(OOD) samples, the energy score *E*(*x*) should be maximized to exceed a specific threshold θ. The energy loss for ID and OOD samples can be expressed as:

$${ℒ}_{\text{energy}}=\frac{1}{N_{\text{in}}}\sum_{i=1}^{N_{\text{in}}}\left[\max(0,E(x_{\text{in},i})-m_{\text{in}}{)}^{2}\right]+\frac{1}{N_{\text{out}}}\sum_{j=1}^{N_{\text{out}}}\left[\max(0,m_{\text{out}}-E(x_{\text{out},j}){)}^{2}\right].$$ (10)

where: $N_{\text{in}}$ is the total number of ID samples in the training set. $N_{\text{out}}$ is the total number of auxiliary OOD samples. $E(x_{\text{in},i})$ is the energy score of the *i*-th ID sample [27]. $E(x_{\text{out},j})$ is the energy score of the *j*-th OOD sample. $m_{\text{in}}$ and $m_{\text{out}}$ are the predefined margins for ID and OOD samples, respectively. The first term penalizes ID samples whose energy scores exceed the margin $m_{\text{in}}$. This ensures that the energy for ID samples remains low. The second term penalizes OOD samples with energy scores below m_out_, ensuring that their energy scores are pushed higher than the threshold.

The total loss for joint classification and open set detection is given by:

$$ℒ={ℒ}_{\text{CE}}+\lambda {ℒ}_{\text{energy}}.$$ (11)

where λ control the relative importance of the energy components.

A threshold θ on *E*(*x*) is used to distinguish ID from OOD samples.

$$\tilde{y}=\left\{ \begin{array}{cc} C+1, & \text{if }E(x)>\theta ,\\ \arg\max_{j\in \{1,\ldots ,C\}}o_{i,j}, & \text{otherwise}. \end{array}\right.$$ (12)

Where *C* + 1 represents the class label for unknown JPs.

Energy-based models(EBMs) are particularly suitable for anomaly detection due to the following reasons:

1. Unified scoring mechanism: EBMs naturally assign low energy (i.e., high compatibility) to in-distribution data and high energy to out-of-distribution or anomalous data, providing a unified and interpretable scoring criterion.
2. Model-agnostic integration: EBMs can be integrated with both generative and discriminative models, and can leverage deep neural networks to model complex data distributions.
3. Training stability and generality: Unlike explicit generative models that require likelihood estimation or reconstruction, EBMs allow implicit density modeling without the need to fully specify the output distribution. Enhanced robustness: Empirical studies [4] have shown that EBM-based methods are more robust to adversarial and high-dimensional anomalies than reconstruction- or density-based methods.

## Simulation experiment

### Data acquisition

To evaluate the proposed method for JP-OSR, synthetic datasets were designed to simulate complex electromagnetic environments. Unlike traditional datasets, where jamming and signal phases are carefully isolated to eliminate cross-interference, the generated data here incorporates diverse JPs to emulate practical communication scenarios. This ensures the robustness and generalizability of the model across different JSR and jamming types. The parameter configurations of each synthetic jamming signal are shown in Table 1. In addition to our synthetic data, we incorporate the publicly available Radio Frequency Jamming Dataset from Kaggle, which provides real-world spectral scans collected via commercial WLAN spectral-scan NICs (Qualcomm Atheros QCA9880 or Doodle Labs NM-DB-3U) combined with the JamRF jamming toolkit. This dataset comprises three labeled scenarios-Deceptive Jamming(DJ), Constant Jammer(CJ), and Reactive Jammer(RJ)-across the 2.4/5 GHz bands.

**Table 1:** Jamming signal configurations and parameters.

Jamming type	Frequency Range (MHz)	Bandwidth factors	JSR (dB)	Time-domain properties
CW	10–20	N/A	–4 to 10 (2-dB steps)	N/A
MTJ	10–20	N/A	–4 to 10 (2-dB steps)	N/A
LFM	10–20	Randomized, 0.2–0.8	–4 to 10 (2-dB steps)	N/A
PBNJ	10–20	Narrow band noise	–4 to 10 (2-dB steps)	N/A
PPNJ	10–20	N/A	–4 to 10 (2-dB steps)	Pulse repetition intervals randomized (0.05–0.25 ms)
FHJ	10–20	N/A	–4 to 10 (2-dB steps)	Randomized hop durations
NLFM	10–20	Randomized, 0.2–0.8	–4 to 10 (2-dB steps)	N/A
PCFJ	10–20	N/A	–4 to 10 (2-dB steps)	Randomized pulse durations
TJ	10–20	N/A	–4 to 10 (2-dB steps)	Adaptive sensing and pulsing behavior

**Notes:** This table summarizes the configurations and parameters of the nine types of jamming signals, highlighting their frequency ranges, bandwidth factors, jamming-to-signal ratios (JSR), and time-domain characteristics.

To verify the effectiveness of the proposed method for JP-OSR, for the first three combinations based on the synthetic dataset, we selected six common types out of the nine jamming signals as known jamming signals, and the remaining three as unknown jamming signals. However, only two of the unknown jamming signals were included in the test set each time, while the remaining unknown jamming signal was used to validate the hyperparameters of the energy model, resulting in a total of three experimental groups. For the fourth combination, we first resampled the real-world data to match the format of the synthetic dataset, and then performed mixed training on the combined data. The specific divisions are shown in Table 2.

**Table 2:** Experimental data combination index.

Combination index	Known JPs	Unknown JPs
Combination 1	CW, MTJ, LFM, PBNJ, PPNJ, FHJ	PCFJ, TJ
Combination 2	CW, MTJ, LFM, PBNJ, PPNJ, FHJ	NLFM, TJ
Combination 3	CW, MTJ, LFM, PBNJ, PPNJ, FHJ	NLFM, PCFJ
Combination 4	CW, MTJ, LFM, PBNJ, PPNJ, FHJ, PCFJ, CJ, DJ	TJ, NLFM, RJ

1. Known Jamming Classes

The following six jamming types are designated as known during training: Continuous Wave (CW), Multi-Tone Jamming (MTJ), Linear Frequency Modulation (LFM), Partial Band Noise Jamming (PBNJ), Pulsed Periodic Noise Jamming (PPNJ), and Frequency-Hopping Jamming (FHJ). Additionally, in the fourth combination the real-world dataset’s Deceptive Jamming (DJ) and Constant Jammer (CJ) samples are also included as known classes in training. These represent commonly encountered JPs with distinct frequency and temporal properties.

2. Unknown Jamming Classes

The following three jamming types are excluded during training and considered unknown: Noise Linear Frequency Modulation (NLFM), Pulsed Constant Frequency Jamming (PCFJ), and Tracking Jamming (TJ). Furthermore, the real-world dataset’s Reactive Jammer (RJ) is treated as an unknown class in the fourth combination. These jamming types introduce additional complexity, mimicking realistic scenarios where new or unseen JPs may emerge.

This configuration ensures comprehensive evaluation of the model’s ability to handle diverse scenarios, including both known and unknown interference types. Moreover, since we adopt the energy model as a tool for open set recognition in the later stages, a portion of OOD data is required for auxiliary training during the training phase. This OOD data must not overlap with the nine types of jamming signals. Therefore, we generate random noise with no significant similarity to actual jamming signals, such as Gaussian white noise and colored noise. These noise signals exhibit uniform spectral distribution and are independent of the modulation patterns or spectral characteristics of the target signals, effectively avoiding confusion with known jamming signals.

### Experimental setup and hyperparameters

In all adaptive-threshold experiments we set the temperature *T* = 1.0 in the energy formula, the scaling factor λ=2.0 to define per-class thresholds $\theta_{c}\;=\;\mu_{c}\;+\;\lambda \sigma_{c}$, and the EMA smoothing coefficient α=0.9. We use a confidence cutoff *p*_*i*_>0.9 on the softmax max-score to admit only high-confidence known-class samples for online threshold updates. These hyperparameters were chosen via grid-search on the validation splits (synthesized and real-world) to balance false-reject versus false-accept trade-offs, and held fixed for all “Combination 4” mixed-training experiments. In the SWT framework, the hyperparameter *Blocks* was uniformly set to 4, while in MCS, the hyperparameter ρ was consistently fixed at 1.0.

The F1 score, calculated using Eq (13), incorporates the true negative rate (TNR) and the true positive rate (TPR). In the subsequent experiments, known jamming samples are labeled as positive, while unknown jamming samples are labeled as negative.

$$F1=2\cdot \frac{\text{Precision}\cdot \text{TPR}}{\text{Precision}+\text{TPR}}.$$ (13)

### Open-set recognition performance

**Fig 4:**
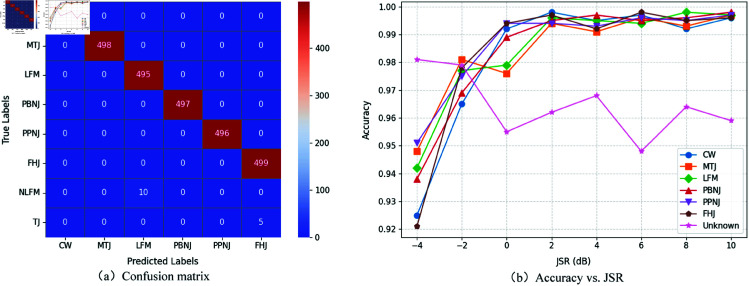
Confusion matrix and accuracy of energy-based JP-OSR.

The confusion matrix for JP-OSR using the energy model is presented in Fig 4a. In this experiment, Combination 1 was selected, with each type of jamming signal comprising 500 test samples in the study. The results indicate that nearly all known jamming samples are correctly classified, with only a negligible number mistakenly identified as unknown. Importantly, no samples were misclassified as other known types of jamming. Furthermore, among the 1,000 samples from the two unknown jamming types, only 15 were misclassified. These results strongly validate the effectiveness and robustness of the proposed method.

Fig 4b illustrates the average accuracy curve of our proposed method across the first three experimental data combinations. As depicted in the figure, when the JSR exceeds –2 dB, the algorithm demonstrates robust recognition accuracy for all known JPs, achieving an average accuracy of 99.5%. At an JSR of -4 dB, while the accuracy is slightly lower, it still maintains a respectable average of 93.4%. Furthermore, the recognition of unknown JPs, despite some variability, consistently surpasses 95%, with an average accuracy of 96.4%. These results underscore the efficacy of our approach for JP-OSR.

To measure and report the computational performance of the proposed energy-based discrimination strategy, we conducted comparative experiments against several representative open-set anomaly detection baselines, including: OpenMax (based on extreme value theory) [18], MSP with ODIN (temperature-scaled logits and input perturbation), Mahalanobis Distance-based detection, G-OpenMax [28], and JC-GAN [25]. To ensure fairness, these methods employed the Spiking Wave Transformer (SWT) as the feature extraction backbone, with parameter settings aligned with defaults or tuning strategies from the original papers. Traditional one-class classifiers, including SVDD and OCSVM [29], as well as the Vision-Transformer-based OSR method ARPL [17] and reconstruction-based methods like VAEs [30], were also included for comprehensive comparison. The evaluation metrics used were TPR, TNR, Precision, and F1-score. Each experiment was repeated five times, and the average results are summarized in Table 3. The results show that the proposed method achieves the best overall performance across all metrics, particularly attaining the highest TNR, Precision, and F1-score. Although ZSL-JPR and JCGAN marginally outperform our method in TPR, ZSL-JPR exhibits sensitivity to outliers, resulting in a conservative threshold selection and lower TNR. Notably, the use of SWT as a unified feature extractor improves the robustness of all compared methods to some extent.

**Table 3:** Comparison between the proposed method and four mainstream methods.

Method	TNR (%)	TPR (%)	Precision (%)	F1-score (%)
SVDD	89.26±0.15	98.68±0.48	93.57±0.28	95.66±0.31
OCSVM	88.25±0.15	99.25 ± 0.35	94.22±0.17	96.33±0.15
ViT-ARPL	94.58±0.14	99.24±0.11	97.25±0.15	98.41±0.26
VAEs	92.44±0.23	99.12±0.18	96.38±0.19	98.31±0.23
Mahalanobis	86.24±0.28	97.58±0.35	92.56±0.27	94.85±0.15
Openmax	79.56±0.25	98.46±0.28	93.25±0.32	94.89±0.27
MSP+ODIN	93.54±0.25	98.59±0.31	96.33±0.23	97.65±0.25
G-Openmax	78.54±0.36	95.63±0.33	95.25±0.30	95.03±0.29
ZSL-JPR	88.23±0.27	** 99.85±0.35 **	93.46±0.37	95.45±0.32
JCGAN	93.62±0.25	99.58±0.36	96.65±0.35	98.43±0.28
Ours	** 95.36±0.15 **	99.48±0.25	** 99.23±0.19 **	** 99.35±0.16 **

To determine whether the observed performance differences are statistically significant, we performed paired t-tests on the F1-scores obtained from the five runs. The p-values for comparisons between the proposed method and each baseline are all less than 0.01, indicating that the improvements are statistically significant at the 99% confidence level.

Further comparative analysis reveals the reasons for the observed advantages. First, the SWT captures both temporal dynamics and frequency structures within a biologically-inspired spiking framework, allowing better feature representation even under low JSR conditions or in highly dynamic signal environments. Second, the proposed energy-based discrimination aggregates confidence scores across all output logits, creating a more stable decision boundary compared to traditional softmax-based approaches like OpenMax and MSP+ODIN, which are vulnerable when known and unknown distributions overlap. In contrast, methods relying on Mahalanobis distance or Gaussian assumptions (e.g., Mahalanobis, G-OpenMax) suffer when feature distributions deviate from unimodal Gaussian forms, leading to performance degradation. Reconstruction-based methods (e.g., autoencoders, VAEs) rely on the assumption that anomalies cannot be well reconstructed. However, such methods often suffer from generalization issues, where the model may still reconstruct anomalous samples well. Overall, these results confirm that the proposed method not only significantly improves detection performance under both synthetic and real-world scenarios but also establishes a robust and statistically validated framework for open-set jamming signal recognition.

### Computational efficiency and latency

To evaluate the inference efficiency of different open-set recognition methods, we measured their per-sample latency, throughput (in frames per second), and model size. All evaluations were conducted on an NVIDIA RTX 3090 GPU with batch size set to 1, simulating real-time processing scenarios.

Unlike prior works that rely on CNNs (e.g., SVDD, JCGAN) or transformer-based backbones (e.g., ARPL), our method adopts a lightweight SWT coupled with MCS. This design enables efficient frequency-domain feature extraction with reduced computational cost.

As shown in Table 4, our method achieves a favorable balance between latency and throughput, with a significantly smaller model size (17.5 MB) compared to most deep learning-based methods. Although SVDD and OCSVM are slightly faster, they rely on much simpler or hand-crafted features and show limited scalability, whereas our method leverages a more expressive feature extractor while maintaining competitive efficiency. These results demonstrate the suitability of our method for real-time RF jamming detection in resource-limited deployment environments.

**Table 4:** Comparison of computational efficiency (Inference-only, batch size = 1).

Method	Latency (ms)	FPS	Model Size (MB)
SVDD (MobileNetV2)	2.5	400	13.6
OCSVM (hand-crafted HOG)	5.0	200	—^*^
JCGAN (GAN + CNN)	8.0	125	30.0
OpenMax (ResNet-50)	7.2	139	102.0
ARPL (ViT-Base/16)	9.0	111	344.0
**Ours (SWT+MCS)**	**6.3**	**159**	**17.5**

**Note:** ^*^OCSVM is a non-parametric kernel method; its model size depends on the number of support vectors and is not directly comparable to deep models.

### Threshold sensitivity analysis

To assess the stability of our self-adaptive threshold under varying jamming-to-signal ratios (JSR) and noise conditions, we performed a grid search over the scaling factor λ∈{1.0,1.5,2.0,2.5} and the EMA coefficient α∈{0.5,0.7,0.9,0.99}. For each (λ,α) pair, we evaluated the *F*_1_-score on test signals at JSR values of –4, –2, 0 and  + 2 dB with additive Gaussian noise at SNR = 20 dB.

**Fig 5:**
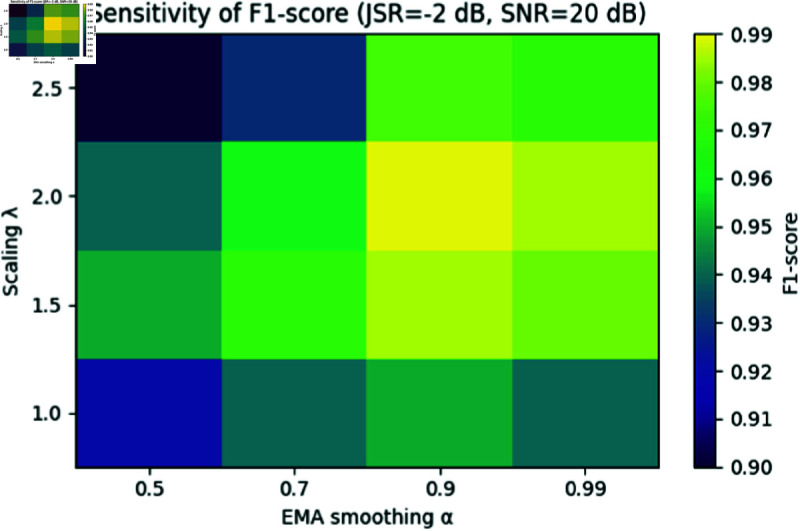
Sensitivity of $F_{1}$-score to adaptive-threshold parameters λ and α at JSR = –2 dB, SNR = 20 dB.

Fig 5 shows a representative heatmap of *F*_1_-scores at JSR = –2 dB and SNR = 20 dB, demonstrating that the region λ∈[1.75,2.25] and α≥0.9 yields consistently high *F*_1_ (> 0.98). Table 5 reports the standard deviation of *F*_1_ across all tested JSR values for each λ (at fixed α=0.9), confirming minimal performance variation when λ=2.0.

**Table 5:** Standard deviation of $F_{1}$-score across JSR ={−4,−2,0,2} dB for each λ (with α=0.9).

λ	$std(F_{1})$
1.0	0.025
1.5	0.015
2.0	0.007
2.5	0.030

These results demonstrate that our adaptive-threshold mechanism is robust: when λ=1.5 and α≥0.9, the *F*_1_-score variation remains below 0.01 across a 6 dB JSR range, confirming stability under diverse interference and noise conditions.

### Ablation study on SWT and MCS

To verify the effectiveness of SWT and MCS in the proposed method, we conduct experiments on three datasets, replacing only one component at a time. When SWT is not used, the CNN network from the literature [25] is used as a substitute. The experimental results are shown in Table 6. As can be seen from the table, when both components are used together, the F1-score is higher. However, when either component is missing, the F1-score decreases slightly, indicating that both SWT and MCS have a positive effect on the model’s performance.

**Table 6:** Ablation experiments on the F1-score of the proposed method.

Dataset	w/o SWT	w/o MCS	w/ SWT and MCS
Combination 1	94.54%	96.85%	**99.43%**
Combination 2	95.68%	96.26%	**98.82%**
Combination 3	96.21%	97.97%	**99.65%**
Average	95.47%	96.82%	**99.30%**

The key hyperparameters in SWT are the number of blocks (denoted as *Blocks*), tested within the range [2, 4, 8], while in MCS, the primary hyperparameter is the hypersphere radius (denoted as ρ), tested within [0.1, 2.0]. When jointly optimized in Combination 1–3, the best F1-score was achieved with *Blocks* = 4 and ρ=1.0. The experimental results clearly indicate that excessive block partitioning (*Blocks* > 4) significantly impairs local anomaly detection performance, while an excessively large hypersphere radius (ρ>>1.0) adversely affects sensitivity to tail classes. Optimal system performance is achieved only through careful parameter tuning, with the best results obtained at *Blocks* = 4 and ρ=1.0, which effectively balances these competing requirements.

## Conclusion, limitations, and future work

The proposed framework addresses critical limitations in existing JP-OSR methods by introducing key innovations in feature extraction, classification, and open-set detection. The SWT enhances time-frequency representations through spiking neuron dynamics, effectively capturing the complex characteristics of jamming signals. Meanwhile, the MCS ensures optimal hyperspherical alignment of class prototypes, thereby improving classification boundaries. Additionally, the energy-based model offers a reliable mechanism for detecting unknown JPs by dynamically adjusting the energy threshold. Compared to traditional approaches, this framework demonstrates superior adaptability to open-set problems, especially in challenging real-world environments like JPR. It provides a robust and scalable foundation for dynamic, high-dimensional tasks, effectively distinguishing between known and unknown JPs.

While the proposed method demonstrates strong performance across both synthetic and real-world jamming scenarios, several limitations should be acknowledged to provide a balanced perspective.

First, under extremely low JSR conditions (e.g., <–4 dB), the spectral signature of jamming signals becomes heavily masked by noise, which may reduce the model’s ability to distinguish unknown jamming patterns. Although the current feature extraction strategy using SWT and MCS mitigates some of this degradation, further enhancements, such as adaptive time-frequency windowing or signal enhancement pre-processing, are worth exploring.

Second, although our SWT + energy model already meets real-time requirements on high-end hardware, its computational complexity may still be prohibitive for ultra-low-power or deeply embedded platforms. Future work will explore model compression techniques such as structured pruning, weight quantization (e.g. 8-bit or even binary networks), and knowledge distillation to create lighter, faster variants without sacrificing detection accuracy.

Third, We note that the present energy-based open-set rule relies on the empirical observation that unseen jamming types tend to induce higher aggregate energy scores than known patterns. In practice, a highly adaptive adversary could craft low-power or spectrally masked jamming signals whose energy footprints overlap those of known classes, violating this assumption. To address such cases, future extensions could integrate complementary cues-such as temporal spike statistics, multi-band correlation features, or learned density models-into the decision rule. Adversarial training with simulated low-energy jammers and robust threshold adaptation (e.g. via reinforcement learning) would further harden the system against stealthy interference.

Finally, while this study evaluates a wide range of synthetic and public real-world data, domain adaptation or continual learning techniques could further enhance the model’s generalization to emerging jamming threats without requiring full retraining.

Building on this framework, future work will explore the integration of additional techniques, such as reinforcement learning, to further enhance the system’s adaptability in highly dynamic environments. Additionally, the extension of this model to multi-modal recognition tasks, such as combining JPs detection with other types of interference, could provide valuable insights for complex real-time applications. We also plan to investigate the use of more advanced energy-based models and deeper feature fusion strategies to further improve detection accuracy and robustness in OSR.
